# Sponge symbioses between *Xestospongia deweerdtae* and *Plakortis* spp. are not motivated by shared chemical defense against predators

**DOI:** 10.1371/journal.pone.0174816

**Published:** 2017-04-18

**Authors:** Micah Jaarsma Marty, Jan Vicente, Benjamin L. Oyler, Allen Place, Russell T. Hill

**Affiliations:** 1 Department of Biology and Marine Biology, Center for Marine Science, University of North Carolina Wilmington, Wilmington, North Carolina, United States of America; 2 Institute of Marine and Environmental Technology, University of Maryland Center for Environmental Science, Baltimore, Maryland, United States of America; University of Genova, ITALY

## Abstract

The recently described epizoic sponge-sponge symbioses between *Xestospongia deweerdtae* and two species of *Plakortis* present an unusual series of sponge interactions. Sponges from the genus *Plakortis* are fierce allelopathic competitors, rich in cytotoxic secondary metabolites, and yet *X*. *deweerdtae* flourishes as an epizoic encrustation on *Plakortis deweerdtaephila* and *Plakortis symbiotica*. Our objective in this study was to evaluate the hypothesis that *X*. *deweerdtae* grows epizoic to these two species of *Plakortis* due to a shared chemical defense against predators. We collected free-living individuals of *X*. *deweerdtae* and symbiotic pairs from a wide geographical range to generate crude organic extracts and a series of polarity fractions from sponge extract. We tested the deterrency of these extracts against three common coral reef predators: the bluehead wrasse, *Thalassoma bifasciatum*, the Caribbean sharpnose puffer, *Canthigaster rostrata*, and the white spotwrist hermit crab, *Pagurus criniticornis*. While the chemical defenses of *P*. *deweerdtaephila* and *P*. *symbiotica* are more potent than those of *X*. *deweerdtae*, all of the sponge species we tested significantly deterred feeding in all three generalist predators. The free-living form of *X*. *deweerdtae* is mostly defended across the region, with a few exceptions. The associated form of *X*. *deweerdtae* is always defended, and both species of *Plakortis* are very strongly defended, with puffers refusing to consume extract-treated pellets until the extract was diluted to 1/256× concentration. Using diode-array high performance liquid chromatography (HPLC) coupled with high-resolution mass spectrometry (LC-MS/IT-TOF), we found two secondary metabolites from *P*. *deweerdtaephila*, probably the cyclic endoperoxides plakinic acid I and plakinic acid K, in low concentrations in the associated—but not the free-living—form of *X*. *deweerdtae*, suggesting a possible translocation of defensive chemicals from the basibiont to the epibiont. Comparing the immense deterrency of *Plakortis* spp. extracts to the extracts of *X*. *deweerdtae* gives the impression that there may be some sharing of chemical defenses: one partner in the symbiosis is clearly *more defended* than the other and a small amount of its defensive chemistry may translocate to the partner. However, *X*. *deweerdtae* effectively deters predators with its own defensive chemistry. Multiple lines of evidence provide no support for the shared chemical defense hypothesis. Given the diversity of other potential food resources available to predators on coral reefs, it is improbable that the evolution of these specialized sponge-sponge symbioses has been driven by predation pressure.

## Introduction

Sponges interact with neighboring benthic organisms in a wide diversity of ways, ranging from mutually beneficial to mutually antagonistic interactions (reviewed by Wulff [1]). Many studies have explored these interactions, documenting cases of allelopathic competition [2–4], collaborative mutualism [5,6], and varying degrees of proximal association [7–9]. These associations may enhance sponge survivorship through increased structural support or protection from predators [5,6,9–11], although sponges sometimes harm their associates [2,3,12,13]. One kind of interaction, epizoism, involves one organism (the epibiont) growing on top of another (the basibiont) without the antagonistic interaction of smothering. The first formal description of sponge-sponge epizoism was from temperate marine caves, where Rützler observed dense communities of epizoic marine sponges [7]. There are now many reports of epizoic sponge associations, although the causes and consequences of the associations are not always explored (e.g. [14–17]).

One sponge-sponge interaction that has been particularly well explored is the case of *Amphimedon erina* growing epizoic to *Geodia vosmaeri*. First reported by Engel and Pawlik [3], these sponge genera represent different approaches to chemical defenses: the Caribbean species of *Geodia* are consistently palatable and preferentially preyed upon, while predators refuse to consume *Amphimedon* spp. [6,18–21]. Given the different chemical defense status of each participant in the symbiosis, Engel and Pawlik [3] proposed that *A*. *erina* might protect *G*. *vosmaeri* by providing a layer of chemically defended tissue. Wilcox *et al*. went on to survey the distribution and abundance of the interaction, and also collect morphological data about each sponge participant [22]. Using gel-screen feeding assays in the field and choice experiments in the laboratory with whole tissue offered to sea stars, Ramsby *et al*. confirmed that *A*. *erina* is chemically defended against predators and *G*. *vosmaeri* is not [11]. These results support the hypothesis that one sponge shares its chemical defenses with another through the symbiosis. In addition, growth experiments suggested that *G*. *vosmaeri* provides vertical habitat for *A*. *erina* to grow, protecting the epibiont from sedimentation and the energetic expense of mucous production [11]. In this case, the epizoic association provides clear benefits to each participant.

A more peculiar example of sponge-sponge epizoism is the symbiosis between *Xestospongia deweerdtae* and one of two possible basibionts: *Plakortis deweerdtaephila* or *Plakortis symbiotica* [23,24]. Sponges in the genus *Plakortis* are rich in cytotoxic secondary metabolites [25–29] and have been shown to swiftly kill neighboring organisms (e.g. [2]). It is therefore unusual that any organism would make contact with any species of *Plakortis*, let alone form a long-term partnership. *Xestospongia deweerdtae* was first described as a free-living deep reef sponge from the north shore of Jamaica [30] but the discovery of this symbiosis [23] prompted a new description of polymorphism to account for its associations to *P*. *deweerdtaephila* and *P*. *symbiotica* [24]. Since *X*. *deweerdtae* can survive on its own and the two species of potential basibionts (*P*. *deweerdtaephila* and *P*. *symbiotica*) may be toxic to neighboring organisms, these symbioses provide a unique scenario to explore sponge-sponge interactions. Vicente *et al*. suggest that the symbioses represent mutualism on the basis of four lines of evidence [23]:

(1) These sponges appear to preferentially live together: biogeographic surveys always showed *X*. *deweerdtae* to be more abundant in association than in free-living form (as high as 23 times more abundant) at sites where symbiotic partners *P*. *deweerdtaephila* and/or *P*. *symbiotica* were present [23]. At sites where *Plakortis* spp. are absent, *X*. *deweerdtae* does grow in its free-living form but it is rare. Furthermore, *P*. *deweerdtaephila* and *P*. *symbiotica* have never been found in a free-living form despite considerable survey effort across the Caribbean.

(2) Both *X*. *deweerdtae* and *Plakortis* spp. exhibit smaller spicules in their associated forms compared to free-living forms, and this pattern persists throughout the Caribbean despite environmental variability in bioavailable silica [23,24]. Although no free-living individuals of *P*. *deweerdtaephila* or *P*. *symbiotica* have been documented, Vicente *et al*. compare these basibionts to the closely-related sponge *Plakortis halichondrioides*, which exhibits similar tissue density but presents significantly longer spicules [23]. A consequence of the symbiosis is that both sponges produce relatively small spicules, which may allow each sponge to allocate more energy to other biological activities.

(3) These epizoic pairs represent the most anatomically interlaced sponge-sponge association ever documented. Dissections of symbiotic pairs revealed that *X*. *deweerdtae* not only grows across the surface of its *Plakortis* basibiont, but grows within the mesohyl and even forms deep oscular channels that may increase the pumping capacity of the basibiont [23]. Increased efficiency of water flow may benefit *Plakortis* spp. by reducing the energy expended on pumping water, and direct contact with *Plakortis* mesohyl may provide *X*. *deweerdtae* access to the microbial phyla of the *Plakortis* basibiont.

(4) As a final line of evidence to suggest that the symbiosis is mutualistic, Vicente *et al*. argue that the symbiosis is life-long and does not cause mortality through smothering [23]. Biogeographic surveys have identified young recruits of *P*. *deweerdtaephila* and *P*. *symbiotica* (≤ 5 cm diameter), and at this early life stage the basibiont already has epizoic *X*. *deweerdtae* [23]. Even large adult individuals exhibit healthy tissue despite a layer of *X*. *deweerdtae* on top. Perhaps the best indication that smothering does not occur is that free-living individuals of *X*. *deweerdtae* show no indication of having smothered a basibiont at some point in the past.

In the original description of the symbioses, Vicente *et al*. propose a shared chemical defense hypothesis that one sponge might benefit by receiving a chemical defense against predators from its symbiotic partner [23]. Clear trade-offs between basic life functions and chemical defenses have been established for the Caribbean sponge fauna [4,31,32], most species of which can be organized into one of two categories: palatable sponges, which lack defenses but grow or reproduce fast enough to persist on reefs, and defended sponges, which grow and reproduce slowly but are not attacked by predators [21,33]. These studies provide a reasonable framework to examine a hypothesis of shared defense in a sponge symbiosis. In the case of *A*. *erina* and *G*. *vosmaeri*, knowledge of sponge chemical defenses was able to successfully predict [3] the outcome of further studies [11]. However the chemical defenses of *X*. *deweerdtae*, *P*. *deweerdtaephila*, and *P*. *symbiotica* have not been previously evaluated. We undertook this study to consider the hypothesis that *X*. *deweerdtae* grows in specialized sponge-sponge symbioses with either *P*. *deweerdtaephila* or *P*. *symbiotica* due to a shared chemical defense against predators. The basibiont species are obvious candidates for the hypothetical role of the sponge in each pairing that provides defensive chemistry; as discussed above, many species within the genus *Plakortis* are rich in bioactive secondary metabolites. Pawlik *et al*. found the congeneric *Plakortis angulospiculatus*, *Plakortis halichondrioides*, and *Plakortis lita* to significantly deter fish feeding in laboratory assays with bluehead wrasse, *Thalassoma bifasciatum* [18]. However it is not unprecedented for a fish predator to tolerate *Plakortis* chemicals: Slattery *et al*. recently observed the Caribbean sharpnose puffer, *Canthigaster rostrata*, taking bites on *P*. *angulospiculatus* and used laboratory feeding assays to demonstrate that this predator was not deterred by some crude extracts generated from *P*. *angulospiculatus* tissue [34]. Whether or not *P*. *deweerdtaephila* and *P*. *symbiotica* are chemically defended, it is also possible that *X*. *deweerdtae* may be defended. A related species, *Xestospongia muta*, has been shown to deter predators in laboratory and field assays [35], although its chemical defenses are categorized as ‘variably defended’ because some individuals exhibit defense while others do not [36].

As in the symbiosis between *G*. *vosmaeri* and *A*. *erina*, it would make sense for the exposed sponge that grows on top to provide the chemical defenses, unless either the veneer of tissue is so thin that predators would bite both species at the same time, or the basibiont species produces copious amounts of defensive metabolites that could be translocated to the epibiont. The former situation is not uncommon in this sponge pair; many specimens exhibit only a few millimeters of *X*. *deweerdtae* tissue. The latter situation, of metabolite translocation, would represent an unusual scenario but requires attention due to the extremely high concentrations of secondary metabolites in the tissues of other sponge species in the genus *Plakortis*.

The goal of this study was to examine the chemical defenses of *X*. *deweerdtae*, *P*. *deweerdtaephila*, and *P*. *symbiotica*. We collected samples from a broad biogeographic region in order to consider differences among populations. We sampled both the free-living and the associated forms of *X*. *deweerdtae*, the *Plakortis* basibiont, and a mixture of the two sponge tissues from each symbiotic pair, in equal parts, to approximate the response of a predator whose bite volume captures more than just the epibiont tissue. Finally, we looked for translocation of metabolites by testing different polarity fractions of each sponge type in fish feeding assays and running diode-array high performance liquid chromatography (HPLC) coupled to mass spectrometry (LC-MS) with nominal and high mass accuracy. To demonstrate translocation, we were looking for secondary metabolites that appear in the extracts of the basibiont and the epibiont but are absent from the free-living form of *X*. *deweerdtae*.

We began the study with three criteria for the acceptance of the shared chemical defense hypothesis. (1) One of the partners in the symbiosis must be consistently defended against predators. (2) One of the partners in the symbiosis must be consistently palatable to predators. This second criterion could be met if the free-living form of *X*. *deweerdtae* is palatable even if the associated form is defended, a potential result of metabolite translocation from the *Plakortis* basibiont to the associated *Xestospongia* epibiont. (3) If the palatable sponge is the associated epibiont *X*. *deweerdtae*, the 1:1 mix of epibiont:basibiont tissue must be deterrent to predators.

## Results

### Assays with crude extracts at volumetric concentration

**Fig 1:**
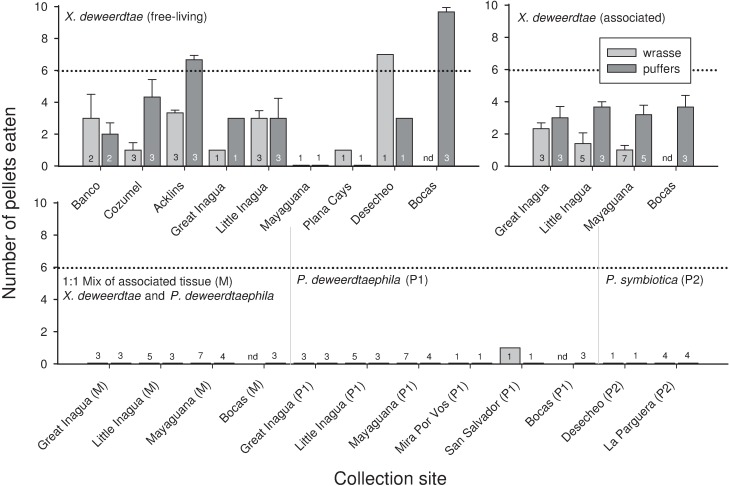
Results of feeding assays with bluehead wrasse (*Thalassoma bifasciatum*) and Caribbean sharpnose puffers (*Canthigaster rostrata*), in which crude organic tissue extracts from sponges, incorporated at a 1× volumetric concentration into artificial food pellets, were offered to each species of fish. Bars indicate the mean number of pellets eaten (out of 10 pellets offered) for multiple individuals of the given sponge at the given site. Error bars show standard error. In every case, 10/10 control pellets were consumed. The dotted line indicates a threshold for statistical significance as determined by a modified Fisher’s Exact Test; a sample is significantly deterrent (*p* < 0.05) if 6 or fewer pellets are consumed [44]. The horizontal axis specifies the geographic location from which the samples were collected, and the number of replicate individual sponges tested from each site is shown as a number overlaid or on top of the bar. nd = no data collected. Tabulated data are archived in S1 Table.

**Fig 2:**
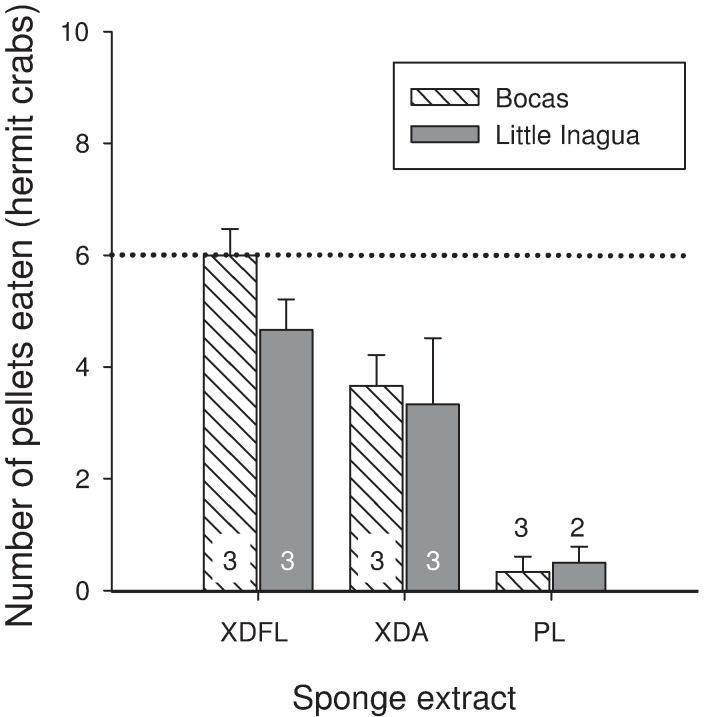
Results of feeding assays with white spotwrist hermit crabs (*Pagurus criniticornis*), in which crude organic tissue extracts from sponges, incorporated at a 1× volumetric concentration into artificial food pellets, were offered to hermit crabs. Bars indicate the mean number of pellets eaten (out of 10 pellets offered) for multiple individuals of the given sponge at the given site. Error bars show standard error. In every case, 10/10 control pellets were consumed. The dotted line indicates a threshold for statistical significance as determined by a modified Fisher’s Exact Test; a sample is significantly deterrent (*p* < 0.05) if 6 or fewer pellets are consumed [44]. The horizontal axis specifies species and growth form used to generate each extract: XDFL = *Xestospongia deweerdtae* free-living; XDA = *X*. *deweerdtae* associated; PL = *Plakortis deweerdtaephila*. The number of replicate individual sponges tested from each site is shown as a number overlaid on the bar. Tabulated data are archived in S1 Table.

In feeding experiments with three model predators, the crude extracts from *Xestospongia deweerdtae* (free-living and associated), *Plakortis deweerdtaephila*, and *Plakortis symbiotica* yielded similar results: most extract-treated food pellets were consumed significantly less than controls (Figs 1 and 2). For free living individuals of *X*. *deweerdtae*, the mean number of treated food pellets eaten by both fish species (puffers and wrasse) from each of the 9 collection sites was significantly less than controls, with three exceptions: Acklins Island (μ = 6.67/10) and Bocas del Toro (μ = 9.67/10) for puffers, and Desecheo (μ = 7/10) for wrasse (Fig 1). Pellets treated with extract of free-living *X*. *deweerdtae* from Bocas del Toro and Little Inagua were also offered to hermit crabs, and the mean number of pellets eaten was less than or equal to 6, although one individual extract from Bocas del Toro was eaten by 7/10 hermit crabs (Fig 2). Extract-treated pellets generated from the associated form of *X*. *deweerdtae* were also consumed significantly less than controls by both fish predators (Fig 1) and by hermit crabs (Fig 2). The 1:1 Mix of *X*. *deweerdtae* and *P*. *deweerdtaephila* was consistently and completely rejected by both fish species, with zero pellets eaten in 28 assays (Fig 1). The same pattern of complete rejection continued for *P*. *deweerdtaephila* extracts, where only 1 pellet was eaten in 1 assay (with an extract from San Salvador, eaten by a wrasse) out of 32 assays for the two fish species (Fig 1). In assays with hermit crabs, *P*. *deweerdtaephila* was also significantly deterrent (Fig 2). We tested extracts from *P*. *symbiotica* collected in Puerto Rico, and consistent with the congener *P*. *deweerdtaephila*, all extracts significantly deterred feeding by both fish species (Fig 1).

### Serial dilution assays

**Fig 3:**
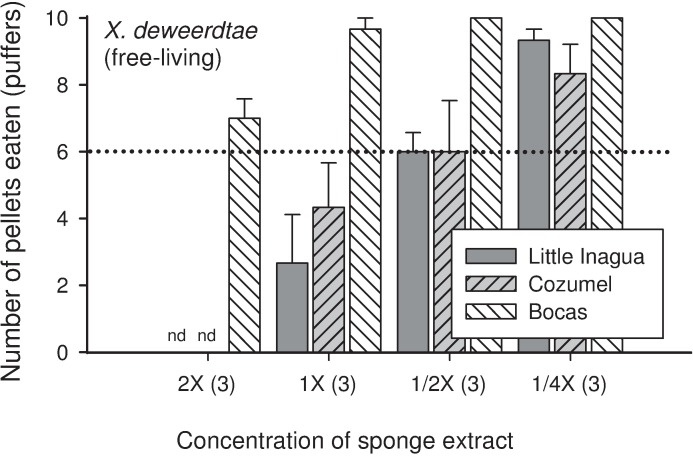
Results from serial dilution feeding assays with Caribbean sharpnose puffers (*Canthigaster rostrata*), using tissue extract from the free-living form of *Xestospongia deweerdtae* from three geographic locations. Horizontal axis shows a progression from concentrated (2×) to dilute (1/4×), and parentheses indicate the number of replicate individual sponges tested. Vertical axis and other details as described for Fig 1.

**Fig 4:**
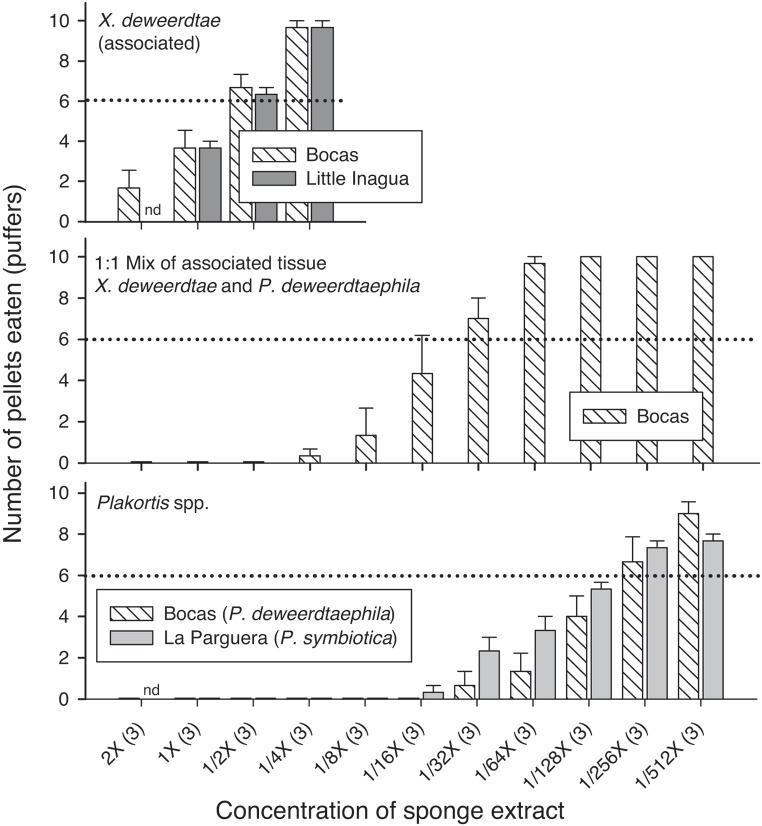
Results from serial dilution feeding assays with Caribbean sharpnose puffers (*Canthigaster rostrata*), using tissue extract from the associated form of *Xestospongia deweerdtae* (top panel), a 1:1 Mix of tissue from both sponges in the association (middle panel), and *Plakortis* spp. (bottom panel). Horizontal axis shows a progression from concentrated (2×) to dilute (1/512×), and parentheses indicate the number of replicate individual sponges tested. Vertical axis and other details as described for Fig 1.

A subset of the sponge extracts was tested at higher and lower concentrations with sharpnose puffers as a model predator. Food pellets containing the 1× volumetric concentration of crude extract from the free-living form of *X*. *deweerdtae* collected at Little Inagua and Cozumel were consumed significantly less than controls, but extracts from Bocas del Toro at the same concentration were not different from controls (Fig 3). A more concentrated version of the free-living *X*. *deweerdtae* extract from Bocas del Toro at 2× was also not significantly different from controls, while the extracts from Little Inagua and Cozumel remained significantly different from controls at 1/2×; pellets from these sites had to be diluted to 1/4× concentration before they were accepted by more than 6/10 puffers on average (Fig 3). For the associated form of *X*. *deweerdtae* from all sites, food pellets treated with extract at a 1× volumetric concentration were consumed significantly less than controls by both fish species (Fig 1). However, when extracts from Bocas del Toro and Little Inagua were diluted to 1/2× concentration and fed to puffers, pellets were consumed at threshold levels that were not significantly different from controls (Fig 4). The 1:1 Mix of *X*. *deweerdtae* and *P*. *deweerdtaephila* tissue significantly deterred both fish predators at 1× concentration (Fig 1) and the extracts from Bocas del Toro required a dilution to 1/32× before puffers would consume more than 6/10 pellets on average (Fig 4). *Plakortis deweerdtaephila* from Bocas del Toro and *P*. *symbiotica* from La Parguera were also significantly deterrent at 1× for both fish predators (Fig 1) and required a dilution to 1/256× before puffers would consume more than 6/10 pellets on average (Fig 4).

### Fractionation assays

**Fig 5:**
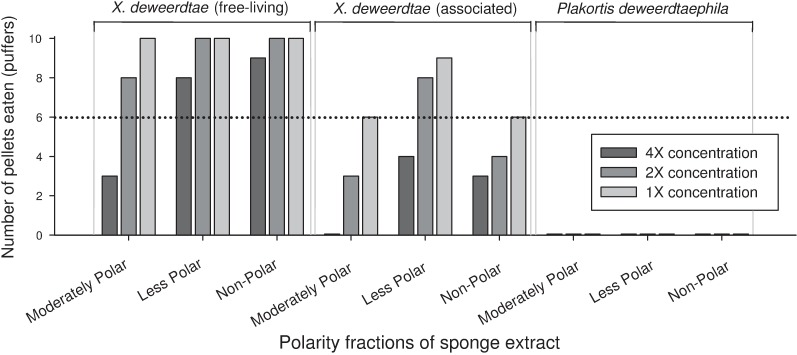
Results from feeding assays in which pellets containing different polarity fractions of sponge extract were offered in serial dilutions to Caribbean sharpnose puffers (*Canthigaster rostrata*). The horizontal axis indicates polarity fractions, and different shades on bars represent different concentrations of the extract fraction. Vertical axis and other details as described for Fig 1.

Sponge tissue samples from *X*. *deweerdtae* (both the free-living and associated forms) and *P*. *deweerdtaephila* collected in Bocas del Toro were each extracted into 3 polarity fractions: moderately polar, less polar, and non-polar. Each extract was tested at 4×, 2×, and 1× concentrations in feeding assays with puffers. None of the fractions of free-living *X*. *deweerdtae* were consumed significantly less than controls except for the 4× concentration of the moderately polar fraction (Fig 5). In contrast, most fractions of associated *X*. *deweerdtae* were consumed significantly less than controls, with the exceptions of 2× and 1× concentrations of the less polar fraction (Fig 5). For *P*. *deweerdtaephila*, all concentrations of all fractions were completely rejected by puffers, with zero pellets eaten (Fig 5).

### Determination of secondary metabolites using diode-array high performance liquid chromatography (HPLC) coupled to mass spectrometry (LC-MS) with nominal and high mass accuracy

**Fig 6:**
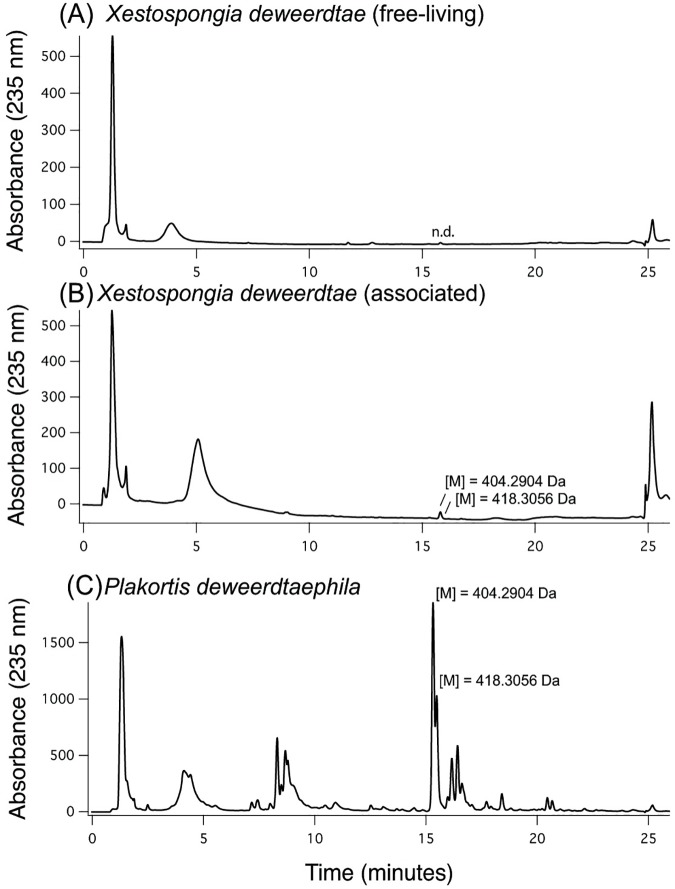
Comparative high performance liquid chromatography (HPLC) chromatograms of crude extracts of sponge tissue dissolved in MeOH (UV absorbance at 235 nm). Neither Main Compound was detected in crude extracts of free-living *Xestospongia deweerdtae*, but both appear in crude extracts of associated *X*. *deweerdtae*, and *Plakortis deweerdtaephila*. Peaks labeled with mass values from subsequent experiment by TOF-MS. Mass spectra and UV absorbance are archived in S1 Fig.

In initial nominal mass accuracy LC-DAD-MS experiments, two peaks at λ max. 235nm around ~15 min were observed to be consistent between extracts from *X*. *deweerdtae* and *P*. *deweerdtaephila*: [M] = 404.2904 Da (determined in a subsequent experiment by TOF-MS; hereafter Main Compound 1) and [M] = 418.3056 Da (determined in a subsequent experiment by TOF-MS; hereafter Main Compound 2) (Fig 6). The mass measurement errors for Main Compounds 1 and 2, when compared against the exact masses of plakinic acids I and K, were -5.7 ppm and -6.5 ppm respectively. On average, Main Compound 1 represented 11.52% of the crude extract in *P*. *deweerdtaephila* and 0.06% of the crude extract in *X*. *deweerdtae*, while Main Compound 2 represented 2.09% of the crude extract in *P*. *deweerdtaephila* and 0.02% of the crude extract in *X*. *deweerdtae* (Table 1). Neither peak from the two Main Compounds was observed in the chromatogram for the free-living form of *X*. *deweerdtae* (Fig 6).

**Table 1:** Proportional masses of Main Compounds from crude sponge extract, λ max. 235, using LC-DAD-MS.

Sponge sample	Crude Extract	Main Compound 1*		Main Compound 2^#^	
	(mg)	(mg)	% of crude	(mg)	% of crude
*Plakortis deweerdtaephila*	100	2.592	**2.592**	0.685	**0.685**
	68.4	9.570	**13.992**	1.717	**2.510**
	40.4	7.262	**17.976**	1.245	**3.081**
*Xestospongia deweerdtae* (associated)	52.2	0.015	**0.029**	0.002	**0.004**
	33.6	0.018	**0.054**	0.007	**0.020**
	29.8	0.028	**0.095**	0.006	**0.020**
*Xestospongia deweerdtae* (free-living)	48.0				
	40.1	NO PEAK		NO PEAK	
	57.6				

*mass [M] = 404.2904 Da determined by a subsequent experiment using TOF-MS.

^#^mass [M] = 418.3056 Da determined by a subsequent experiment using TOF-MS.

## Discussion

In this study, we evaluated the hypothesis that *Xestospongia deweerdtae* grows in specialized sponge-sponge symbioses with either *Plakortis deweerdtaephila* or *Plakortis symbiotica* due to a shared chemical defense against predators. We collected free-living individuals of *X*. *deweerdtae* and symbiotic pairs from a wide geographical range and tested the chemical defenses of these sponges against three common coral reef predators in feeding assays with crude extracts, serial dilutions of extracts, and a series of polarity fractions from sponge extract. The multiple lines of evidence we gathered provide no support for the hypothesis that this symbiosis is driven by shared chemical defenses. To attribute the symbiosis to a shared defense would require at least one of the partners in symbiosis be consistently palatable to predators. While the chemical defenses of *P*. *deweerdtaephila* and *P*. *symbiotica* appear more potent than those of *X*. *deweerdtae*, all of the sponge species we tested significantly deterred feeding by all three generalist predators. The free-living form of *X*. *deweerdtae* is mostly defended across the region, with a few exceptions. The associated form of *X*. *deweerdtae* is always defended, and both species of *Plakortis* are very strongly defended, with puffers refusing to consume extract-treated pellets until the extract was diluted to 1/256× concentration. *Plakortis deweerdtaephila* is so rich in secondary metabolites that we even found the major constituents from *P*. *deweerdtaephila* at very low concentrations in associated *X*. *deweerdtae* tissue. Comparing the immense deterrency of *Plakortis* spp. extracts to the extracts of *X*. *deweerdtae* gives the impression that there may be some sharing of chemical defenses: one partner in the symbiosis is clearly *more defended* than the other and a small amount of its defensive chemistry may translocate to the associated epibiont. However, the chemical defenses in each sponge species deter feeding in fish and arthropod predators, and given the diversity of other potential food resources on coral reefs it is improbable that the evolution of these specialized sponge-sponge symbioses has been driven by predation pressure.

The free-living form of *X*. *deweerdtae* varies slightly in deterrency across its geographic range, with assay fishes rejecting pellets treated with the crude extract of some individuals while consuming pellets treated with the crude extracts of other individuals. This variability can be seen clearly in the serial dilution assays with puffers, where samples from Bocas remain palatable even when concentrated to 2×, but samples from Little Inagua and Cozumel require a 1/4× dilution before they are palatable (Fig 3). Some common Caribbean sponges, such as the chicken liver sponge *Chondrilla caribensis*, the giant barrel sponge *Xestospongia muta*, and the pink vase sponge *Niphates digitalis*, exhibit an exaggerated form of this pattern, where even within the same population, individuals will vary dramatically in their levels of chemical defense [35–37]. It is possible that intra-specific variation in the trait of chemical defense may allow a sponge species to avoid predation: as predators incur handling costs by sampling and rejecting defended individuals, they may shift their foraging effort to other resources and ignore the variably defended species [36]. In any case, the free-living form of *X*. *deweerdtae* is defended at so many of the sites throughout the region that it fails to meet the criteria of a ‘variably defended’ sponge (*sensu* Loh and Pawlik [21]) and as a morphotype of the species, it is properly categorized as defended. Fractionation assays with tissue from Bocas suggest that any deterrent compounds are in the moderately polar fraction; however the Bocas tissue samples were not defended as crude extracts and may not indicate the defenses that cause fishes to reject this species from the sites where it is defended. The associated form of *X*. *deweerdtae* is consistently defended across its range, although a dilution to 1/2× puts it just above threshold palatability. As with the free-living form, the moderately polar fraction of associated *X*. *deweerdtae* was deterrent in fractionation assays with puffers. Distinct from the free-living form, however, the non-polar fraction was also deterrent. It is possible that the non-polar activity comes from Main Compounds 1 and 2. Overall, extracts from *X*. *deweerdtae*, in both the free-living and associated forms, deter feeding by fishes and hermit crabs. We classify this species as a chemically defended sponge.

The two species of *Plakortis* basibionts showed remarkable deterrency. Crude extracts of both species significantly deterred feeding in puffers and wrasse, and extract-treated pellets of *P*. *deweerdtaephila* were also rejected by hermit crabs. Serial dilutions provided striking results: puffers rejected treated pellets until dilution reached 1/256× (Fig 4). While there may be multiple defensive roles for secondary metabolites, both species of *Plakortis* examined in our study clearly have more chemical defense than necessary to deter predators. We also tested a mixture of equal parts tissue from each sponge in a given sponge pair. The bite volume of most spongivorous fishes is large enough to capture not just tissue from the epibiont *X*. *deweerdtae*, but also the *Plakortis* spp. basibionts, prompting the following question: if the epibiont tissue had been palatable, would fishes still avoid it because a portion of the bite might include the basibiont? The results from assays with the tissue of individual sponges indicate that both the epibiont and the basibiont are chemically defended, and—as one would expect—the combination was also deterrent, requiring substantial dilution (1/32×) before it was accepted as palatable in dilution assays with puffers. In sum, the *Plakortis* basibionts are remarkably deterrent, and even small amounts of the extract demonstrated significant anti-predator activity.

In search of the metabolites responsible for deterrency, we used diode-array high performance liquid chromatography (HPLC) coupled mass spectrometry (LC-MS) with nominal and high mass accuracy to examine tissue extracts. Among the samples, we found two major peaks, Main Compound 1 and Main Compound 2, in *P*. *deweerdtaephila* and the associated form of *X*. *deweerdtae*. Main Compound 1 was highly concentrated in *P*. *deweerdtaephila*, representing nearly 18% of the crude extract from one sample. The same compound only represented fractions of percentages of the crude extract of the associated *X*. *deweerdtae*. Similar patterns were observed for Main Compound 2, where again a greater amount was found in *P*. *deweerdtaephila* than the associated *X*. *deweerdtae*. The disproportionately high amounts of these Main Compounds in *P*. *deweerdtaephila* relative to the associated *X*. *deweerdtae* suggest that the compounds may be generated in *P*. *deweerdtae* and translocated to its epibiont, the associated *X*. *deweerdtae*. Perhaps most notable was the absence of both peaks from the HPLC chromatogram of the free-living form of *X*. *deweerdtae*, which supports the possibility of translocation. Variations in the amount of Main Compounds 1 and 2 were observed among the individuals of each species, probably as a result of sponge morphology and the dissection procedure. Previous studies have shown that concentrations of secondary metabolites may vary among different tissues of the sponge (e.g., [38]), and it is possible that these differences caused the variation in compound concentrations that we detected.

**Fig 7:**
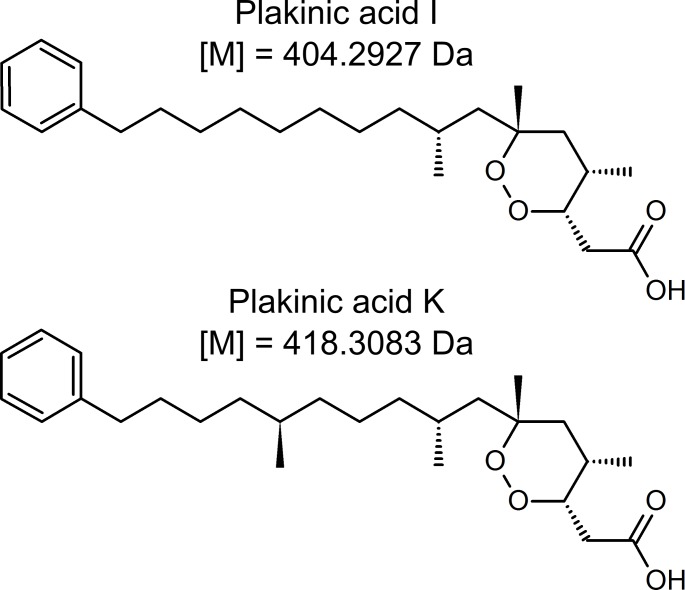
Chemical structures of plakinic acids I (C_25_H_40_O_4_) and K (C_26_H_42_O_4_) [38,39]. The exact monoisotopic masses for these metabolites are consistent with the neutralized measured masses of Main Compounds 1 ([M] = 404.2904 Da, -5.7 ppm) and 2 ([M] = 418.3056 Da, -6.5 ppm).

We suspect that the Main Compounds we detected are plakinic acids, a class of cyclic endoperoxides. Main Compounds 1 and 2 exhibited the same masses as plakinic acids I and K, respectively (Fig 7), both of which were originally isolated and described from *P*. *deweerdtaephila* [39,40]. Extracts of *P*. *deweerdtaephila* run on thin layer chromatography (TLC) plates and sprayed with p-anysaldehyde also indicated the presence of plakinic acids (see S1 Text). On the basis of the TOF-MS data and the TLC results, we believe that the Main Compounds in this study are plakinic acids I and K. After all, these plakinic acids were originally derived from *P*. *deweerdtaephila* and their UV absorbance (235nm) is the same as our Main Compounds. However, without ^1^H NMR or ^13^C NMR data, it is difficult to ascertain the exact structures of Main Compounds 1 and 2.

Could these compounds be responsible for deterrency and represent a shared chemical defense? While no studies have tested plakinic acids for anti-predator activity and we did not isolate Main Compounds 1 and 2 to test in feeding assays, it is possible that the Main Compounds function in *P*. *deweerdtaephila* as chemical defenses against predators. Plakortide F, a compound from sister taxon *Plakortis angulospiculatus* that also contains endoperoxides, has shown anti-predator activity in feeding assays [34]. Main Compounds 1 and 2 may deter feeding, given their probable structural similarity to Plakortide F, their substantial contribution to the crude extract in *P*. *deweerdtaephila*, and the immense deterrency of the *P*. *deweerdtaephila* crude extract. If they were deterrent, these compounds may also have contributed to the deterrency of the extract of associated *X*. *deweerdtae* through translocation. Both compounds were observed as peaks in the HPLC chromatogram from associated *X*. *deweerdtae* extract, which was deterrent in the non-polar fraction where Main Compounds 1 and 2 occur. By contrast, the same peaks were absent from the HPLC chromatogram of the free-living *X*. *deweerdtae*. Additionally, the non-polar fraction of the extract from the free-living *X*. *deweerdtae* was palatable. While it is unclear how much of the deterrency of the associated *X*. *deweerdtae* extract might be attributed to the Main Compounds, our results suggest the possibility that *P*. *deweerdtaephila* produces excess metabolites that then translocate into the tissue of the epibiont *X*. *deweerdtae*, perhaps functioning as a ‘shared defense.’ It is important to remember that LC-MS, TLC, and the fractionation assays were only conducted with tissue from Panama and these results cannot be generalized with confidence to occurrences of this sponge pair throughout the region. Additionally, these lines of evidence are circumstantial and any conclusions drawn from them should be considered hypothetical. Nevertheless, associating with *P*. *deweerdtaephila* may provide extra non-polar deterrency to *X*. *deweerdtae* through the translocation of metabolites generated in *P*. *deweerdtaephila*.

And yet, extracts from the free-living form of *X*. *deweerdtae* were deterrent to all three model predators employed in this study. *Xestospongia deweerdtae* clearly generates effective anti-predator chemical defenses on its own, without the biochemical assistance of its symbiotic partner. We suspect that the deterrency of *X*. *deweerdtae* is the result of polar metabolites: Chanas and Pawlik conducted feeding assays with different polarity fractions of extract from the congener *Xestospongia muta*, and found that the deterrent activity was in the polar fraction [35]. In this study we detected deterrent activity in the polar fraction of both growth forms, but we were unable to fractionate the extract further to isolate the deterrent component. Whatever metabolites may defend *X*. *deweerdtae* from predation, this species clearly does not require an association with *P*. *deweerdtaephila* or *P*. *symbiotica* to deter predators.

Both blueheads and puffers have been used to study the chemical defenses of sponges in the past (e.g., [33,34]), yet this is the first time that a direct comparison has been made between the chemical defense feeding assay results of these fish species from two distinct orders. The responses of these fishes rarely produced statistically different outcomes in our assays. The minor differences that we do observe are not consistent across samples: for the free-living form of *X*. *deweerdtae*, puffers consumed more treated pellets from Acklins Island than did wrasse, but wrasse consumed more treated pellets from Desecheo than did puffers. The comparison of these two model predators merits further investigation using a broader suite of prey organisms in order to examine differences in fish response.

Our use of a pellet-based feeding assay with hermit crabs generated similar results to our fish-feeding experiments. Although previous studies have compared the feeding of fishes and crabs, our assay designs allow for a direct comparison of results. Pennings *et al*. tested the deterrency of sponge metabolites against *Canthigaster solandri* (closely related to *C*. *rostrata*) and intertidal xanthid crabs from the genus *Leptodius*, and found different predator responses [41]; however, the design of their assays left ambiguity as to whether the different results were actually caused by differences in gustatory perception by the predators or whether the differences were caused by the design of the assay. Waddell and Pawlik evaluated the response of the hermit crab *Paguristes punticeps* to crude extracts generated from a suite of Caribbean sponges [19] that had previously been tested against bluehead wrasse, *T*. *bifasciatum* [18]. Although the assay design between the two studies was slightly different, the results were similar, and Waddell and Pawlik conclude that chemical defenses in sponges are broadly effective against a diversity of predators [19]. Our hermit crab assays shared the same design as our fish-feeding assays, and a direct comparison of the results suggests that the chemical defenses of *X*. *deweerdtae* and *Plakortis* spp. are broadly effective against fish and arthropod predators.

While our use of these three model predators is substantial, it does not exhaustively evaluate the potential predators of this sponge pair, and there may be predators who are not deterred in their feeding by the defensive chemistry of these sponges. An alternative scenario is that defenses in the epizoic *X*. *deweerdtae* protect *P*. *deweerdtaephila* and *P*. *symbiotica* from some surface-feeding predator (like a gastropod or an asteroid). This scenario is improbable, but it is consistent with the observation that neither species of *Plakortis* can be found in a free-living form. Sponges in the genus *Aplysina*, for example, are exceptionally well defended against vertebrate and invertebrate predators [18–20] using brominated tyrosine derivatives as a chemical defense (e.g. [42]). However, cowries in Belize [43] and limpets in Panama (Vicente, *unpublished data*) have recently been observed to feed voraciously on *Aplysina* spp. despite the substantial chemical defenses. In an effort to consider alternative predators of *X*. *deweerdtae* and *Plakortis* spp., we attempted gel-screen feeding assays with a limpet and a dorid nudibranch, but these gastropod predators refused to cooperate under the control conditions.

In summary, a broad biogeographic survey of the chemical defenses in this epizoic sponge symbiosis did not support the shared chemical defense hypothesis. While we found some evidence for the translocation of secondary metabolites from *P*. *deweerdtaephila* to *X*. *deweerdtae*, this probably does not provide a necessary chemical defense to *X*. *deweerdtae*, which already possesses its own chemical defenses. These sponges survive together—perhaps even thrive—in a close symbiotic relationship. But each wields its own chemical defenses to deter fish and arthropod predators.

## Methods

### Ethics statement

Research was conducted under the CONAPESCA permit DAPA/2/06504110612/1608 in Mexico, permit 2010-IC-043 (R-VS-PVS15-SJ-00190-08062010) issued by the Department of Natural Resources in Puerto Rico, fauna collection permit nos. 05 and 07 issued by the Autoridad Nacional del Ambiente (ANAM) and no. SC/A-27-15 issued by the Ministerio de Ambiente de Panamá in Bocas del Toro. Unnumbered scientific permits were provided by the Department of Marine Resources of the Bahamas. None of the species collected for this study are endangered or protected.

Our work with fishes, as model predators in feeding assays, strictly followed the National Institutes of Health’s recommendations in the Guide for the Care and Use of Laboratory Animals. The Institutional Animal Care and Use Committee of the University of North Carolina Wilmington (A1213-015) approved the protocol for *Thalassoma bifasciatum* (bluehead wrasse). The protocol for *Canthigaster rostrata* (sharpnose puffer) was approved by the Institutional Animal Care and Use Committee of the Smithsonian Tropical Research Institute (2015-0715-2018).

### Collection

Sponge tissue was collected from 13 coral reef sites across the Caribbean in the Bahamas, the Mexican Yucatan, Panama, and Puerto Rico (Table 2). A small piece of tissue (<20mL) was taken from each sponge. For pairs of associated *Xestospongia deweerdtae* and *Plakortis deweerdtaephila* or *Plakortis symbiotica*, live tissue was dissected immediately after collection to separate the tissues of each sponge from one another. Extra tissue from each sponge pair was also combined, in equal portions, to form a sample group representing a 1:1 mixture of tissue. Wet tissue for all sample types was frozen at -80°C until extraction or placed immediately into solvents; volume of extracted tissue was measured by volumetric displacement.

**Table 2:** Collection locations for sponge material and assay predators employed in this study. Each collection took place in June or July of the year specified, except for samples from Puerto Rico, which were collected in October.

Location	Site	GPS Coordinates	Year
Bahamas	Acklins Island	22°10.617’N 74°17.367’W	2013
	Great Inagua	21°04.983’N 73°38.917’W	2013
	Little Inagua	21°27.048’N 73°03.404’W	2013
	Mayaguana	22°26.150’N 73°08.083’W	2013
	Mira Por Vos	22°06.052'N 73°32.444'W	2011
	Plana Cays	22°36.270'N 73°32.792’W	2011
	San Salvador	24°02.433'N 74°31.882'W	2011
Mexico	Banco Chinchorro	18°34.894'N 87°25.067'W	2012
	Cozumel	20°22.937'N 87°01.746'W	2012
Panama	Dolphin Rock	9°21.207’N 82°11.132’W	2015
	Hospital Point	9°20.020'N 82°13.140'W	2015
	Punta Caracol	9°22.690'N 82°18.230'W	2015
	STRI Point	9°20.576’N 82°15.489’W	2015
Puerto Rico	Desecheo	18°23.506'N 67°28.558'W	2011
	La Parguera	17°53.297'N 66°59.887'W	2011

### Extraction for general feeding assays

The chemical extraction procedures for general feeding assays were conducted as described by Marty and Pawlik [43]. Briefly, the tissue was agitated vigorously in a 1:1 solvent mixture of dichloromethane (DCM) and methanol (MeOH) for a 6 hr extraction period. The polar and nonpolar fractions of the DCM:MeOH extract were filtered, then dried separate from one another using rotary evaporation at low heat (<40°C) while a subsequent extraction in MeOH was performed. All of the fractions were then combined into one crude organic extract and dried using vacuum concentration, again at low heat (<40°C). Dried extracts were stored under N_2_ gas at -20°C until use in feeding assays.

### Extraction for fractionation assays

Additional tissue samples of *X*. *deweerdtae* (both the free-living and associated forms) and *P*. *deweerdtaephila* collected in Bocas del Toro were extracted using the following procedure in order to examine different polarity fractions of each extract. Centrifuge tubes (50 mL) containing 15 mL of tissue were agitated vigorously during a 4 hr extraction period for each solvent. First, wet tissue was extracted in MeOH, resulting in a moderately polar solvent mixture of H_2_O and MeOH. After removal of this moderately polar extract, the dehydrated tissue was extracted a second time in MeOH, representing a less polar fraction. A third extraction was achieved using chloroform, with the series of extracts representing a series of polarity fractions, progressing from moderately polar to non-polar. Fractions were filtered, then dried using rotary evaporation and vacuum concentration at low heat (<40°C) and stored dry under N_2_ gas at -20°C until use in feeding assays.

### Extraction for LC-MS

Additional tissue samples of *X*. *deweerdtae* (both the free-living and associated forms) and *P*. *deweerdtaephila* collected in Bocas del Toro were extracted using the following procedure in order to run diode-array high performance liquid chromatography coupled with mass spectrometry. Tissue samples were lyophilized, and 500 mg of each sample was extracted four times in a 1:1 solvent mixture of chloromethyl and MeOH. The four extracts were then combined, filtered, and concentrated using rotary evaporation under pressure and low heat (<40°C).

### Feeding assays

The core data in this study were generated using feeding assays that have been designed to isolate the taste of a potential prey item and evaluate the response of a model predator to that taste in a simulated predation event. We follow the principles for chemical defense feeding assays discussed by Marty and Pawlik [44], using two fish species that have been employed previously in these sorts of assays as well as a novel pellet-based hermit crab assay. All experimental protocols involving the use of live vertebrates were approved by the Institutional Animal Care and Use Committees (IACUCs) at the University of North Carolina Wilmington (UNCW) and the Smithsonian Tropical Research Institute (STRI) and strictly followed the National Institutes of Health (NIH) recommendations in the Guide for the Care and Use of Laboratory Animals. Yellow-phase bluehead wrasse, *Thalassoma bifasciatum*, were collected off Key Largo, Florida and immediately shipped airfreight to UNCW by a licensed fish vendor in Florida. For feeding assays conducted at the STRI field station in Bocas del Toro, Panama, we collected the Caribbean sharpnose puffer, *Canthigaster rostrata*, at Hospital Point and the white spotwrist hermit crab, *Pagurus criniticornis*, at STRI Point (Table 2).

Experimental foods were prepared as described by Marty and Pawlik [44]. Extracts, suspended in a minimal volume of MeOH, were reconstituted in a nutritionally appropriate food matrix of sodium alginate and squid mantle at the *volumetric* concentration of the source sponge, then formed into hardened strands in calcium chloride and cut into 4 mm pellets. Control pellets were prepared in the same manner—including the addition of MeOH—but without sponge extract. Pellets from each extract were presented in laboratory feeding assays to each of 10 independent groups of three wrasse. Feeding assays with puffers were run in the same fashion, except fish were held in isolation from one another. Extract-treated pellets were considered rejected if sampled and regurgitated by the fish three times or sampled once and subsequently ignored. We also conducted feeding assays with hermit crabs held individually in 300 mL glass bowls. These hermit crabs forage on coral reef sponges at night, so we conducted hermit crab assays after sundown, although the experimental bowls were illuminated for the duration of experiments. Hermit crabs were offered pellets generated in the same manner as those that were fed to fishes. Since this species of hermit crab is very small (< 2 cm carapace) a pellet represented more than a single bite: in order for a pellet to be scored as accepted, the hermit crab had to pick up the pellet and feed for 60 seconds. If the hermit crab began feeding but discarded the pellet in less than 60 seconds, the pellet was scored as rejected. Also, if the hermit crab held the pellet for 60 seconds but constant spitting was observed as the crab attempted to feed, the pellet was scored as rejected. For all three model predators, a rejection of any treated pellet was followed by the offering of a control pellet. If the criteria for accepting a pellet were met with the subsequent control pellet, the sample could be scored as rejected [44].

### Serial dilution and fractionation assays

To evaluate the magnitude of chemical defense, extract-treated pellets were first offered to puffers at 2× concentration, then serially diluted to 1×, then 1/2× and so on until the extract was fully palatable. In the cases where no data are available for 2× concentration, this is because the extracts were mixed first at 1× before we decided to conduct serial dilution assays. The samples that were extracted in different polarity fractions were first mixed into food at 4× concentration, then serially diluted to 2× and 1× concentrations for feeding assays with puffers. It is possible for metabolites to interact in a summative or synergistic way, and fractionation may remove the interaction effect, so it is important to test each fraction at higher-than-natural concentrations [45].

### Statistical analysis of feeding assay data

Significance of differences in consumption of treated versus control pellets was evaluated using a modified version of Fisher’s exact test in which the marginal totals for control and treated pellets were fixed, treating them both as random samples [44]. Individual extracts were scored as deterrent if six or fewer pellets were consumed (*p* < 0.05), and in the case of a group of extracts (e.g. all *P*. *deweerdtaephila* extracts from Mayaguana that were tested on puffers), the group was considered deterrent if the mean number of pellets eaten was less than or equal to six [36].

### LC-DAD-MS

For initial analytical experiments, samples were suspended in 500 μl of MeOH and 5 μl were injected onto a C8 (LiChrosphere 125mm x 4mm, 5 μm bead size RP-8, Agilent) column at 30°C and subjected to a 0.8 mL / min. 10% to 95% acetonitrile:water gradient over 25 min using a high performance liquid chromatograph (HPLC, Agilent 1100). Metabolite peaks were detected using an Agilent Diode Array Detector (DAD, Model #G1315B) with a micro high-pressure flow cell (G1315B#020; 6 mm pathlength, 1.7 μl volume) over the wavelength range 190 to 950 nm. Based on the UV spectra, the absorption at 235±4 nm was used to detect plakinic acids. The UV absorbance for each UV detectable peak is archived in S1 Fig. The eluate from the DAD detector was fed to the electrospray nozzle of the mass spectrometer (Agilent G1956A SL) for ionization with the following spray chamber conditions using N_2_ as the drying gas: flow rate 10 L / min, pressure 60 psi, temperature 350°C, fragmentor voltage 70 V, capillary voltage 4000 V. Isopropanol (0.01 mL / min) was added post-column to provide appropriate conditions for positive mode ionization. Mass spectra (200–600 *m/z*) were obtained for all UV peaks.

### LC-MS/IT-TOF data acquisition and processing

In order to support mass assignments from nominal mass accuracy experiments, extracts were analyzed using a Prominence LC-20AD binary LC system (Shimadzu Scientific Instruments, Columbia, MD, USA), equipped with an autosampler and diode array detector, and coupled to a LC-MS/IT-TOF mass spectrometer (Shimadzu Scientific Instruments, Columbia, MD, USA). Reversed phase separation was achieved on a Phenomenex (Torrance, CA, USA) Kinetex C8 column (50 mm x 2.1 mm i.d., 2.6 μm particle size). A binary gradient elution program was performed using Optima (Fisher Scientific, Waltham, MA, USA) LC-MS grade water (Solvent A) and Optima (Fisher Scientific, Waltham, MA, USA) LC-MS grade acetonitrile (Solvent B) from 5% Solvent B to 95% Solvent B in 15 minutes. Accurate mass spectra (S1 Fig) were collected in both positive and negative ionization modes, from *m/z* 100–1000, by switching the polarity during a single chromatographic run. Raw data files were processed in LCMSSolution software version 3.7 (Shimadzu Scientific Instruments, Columbia, MD, USA). The exact mass of an extra proton (1.007825 Da) was added to the measured [M-H]^-^ ions, detected in negative ionization mode, to compare molecular weight ([M]) for each Main Compound against previous literature and previous experiments. The error in the measurement was calculated as ([M_measured_]—[M_exact_]) / [M_exact_] and reported in parts per million (ppm). Accurate masses for the Main Compounds were manually compared to theoretical monoisotopic masses for plakinic acids.

## Supporting information

S1 Table Tabulated data of all feeding assay results. (XLSX)

S1 Text Thin-layer chromatography (TLC) of plakinic acids. (PDF)

S1 Fig Comparative mass spectra (top panels) and UV absorbance (bottom panels) from crude extracts of sponge tissue dissolved in MeOH and run on LC-DAD-MS. Results for Main Compound 1 shown in panels A and C; Main Compound 2 is shown in panels B and D.
(TIF)
