# Supplementary material for: Sponge symbioses between Xestospongia deweerdtae and Plakortis spp. are not motivated by shared chemical defense against predators
Source: PLoS One. 2017 Apr 18;12(4):e0174816. doi: 10.1371/journal.pone.0174816 (PMC5395162; doi:10.1371/journal.pone.0174816)
Supplement: S1 Text — (PDF) [file pone.0174816.s002.pdf]

The following supplement accompanies the article

Sponge symbioses between *Xestospongia deweerdtae* and *Plakortis* spp. are not motivated by shared chemical defense against predators

Micah Jaarsma Marty, Jan Vicente, Benjamin L. Oyler, Allen Place, and Russell T. Hill*

*Corresponding Author: hill@umces.edu

PLOS ONE

Supporting Information: Methods & Results for thin-layer chromatography (TLC) of plakinic acids.

Methods

The crude extract from each sponge species, originally suspended in 500 µl of MeOH and previously used for LC-MS analysis, was blotted and dried on dry plates of silica gel for thin-layer chromatography (TLC). The plates were then placed in a 95:5 hexane:EtOAc + 0.1% glacial acetic solvent system for 30 minutes. Plates were dried and sprayed with p-anysaldehyde, then heated at 100 °C until spots were visible. Plakinic acids appear as bright pink spots (Jamison *et al.*, 2016).

Results

Pink spots indicative of plakinic acids were observed in all three extracts of *P. deweerdtaephila* (Fig A). The extracts of *X. deweerdtae* in both the free-living and associated forms lacked the pink spots to indicate the presence of plakinic acids (Fig A).

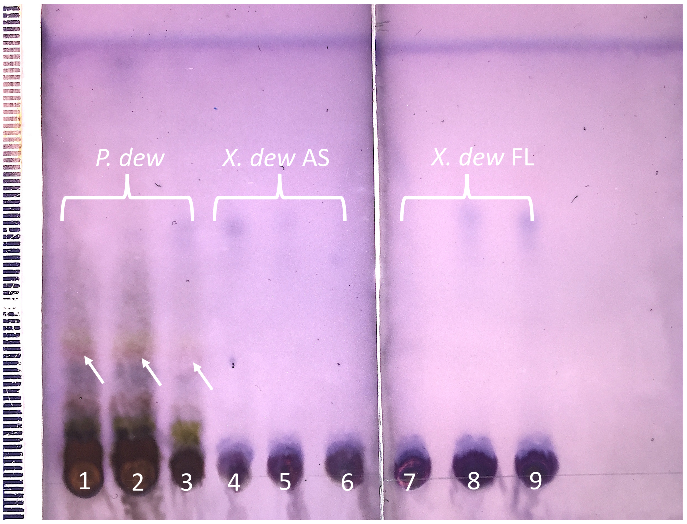

Fig A. Thin-layer chromatography (TLC) plates run with crude extracts from *P. deweerdtaephila* (lanes 1-3), associated *X. deweerdtae* (lanes 4-6) and free-living *X. deweerdtae* (lanes 7-9). Pink spots indicative of plakinic acids appear in lanes 1-3 but not lanes 4-9. Spots are indicated by white arrows.

References

Jamison, MT, Dalisay, DS, & Molinski, TF. Peroxide Natural Products from *Plakortis zyggompha* and the Sponge Association *Plakortis halichondrioides–Xestospongia deweerdtae*: Antifungal Activity against *Cryptococcus gattii*. Journal of Natural Products. 2016;79(3):555-63.
