# Supplementary figures and images for: Sponge symbioses between Xestospongia deweerdtae and Plakortis spp. are not motivated by shared chemical defense against predators
Source: PLoS One. 2017 Apr 18;12(4):e0174816. doi: 10.1371/journal.pone.0174816 (PMC5395162; doi:10.1371/journal.pone.0174816)

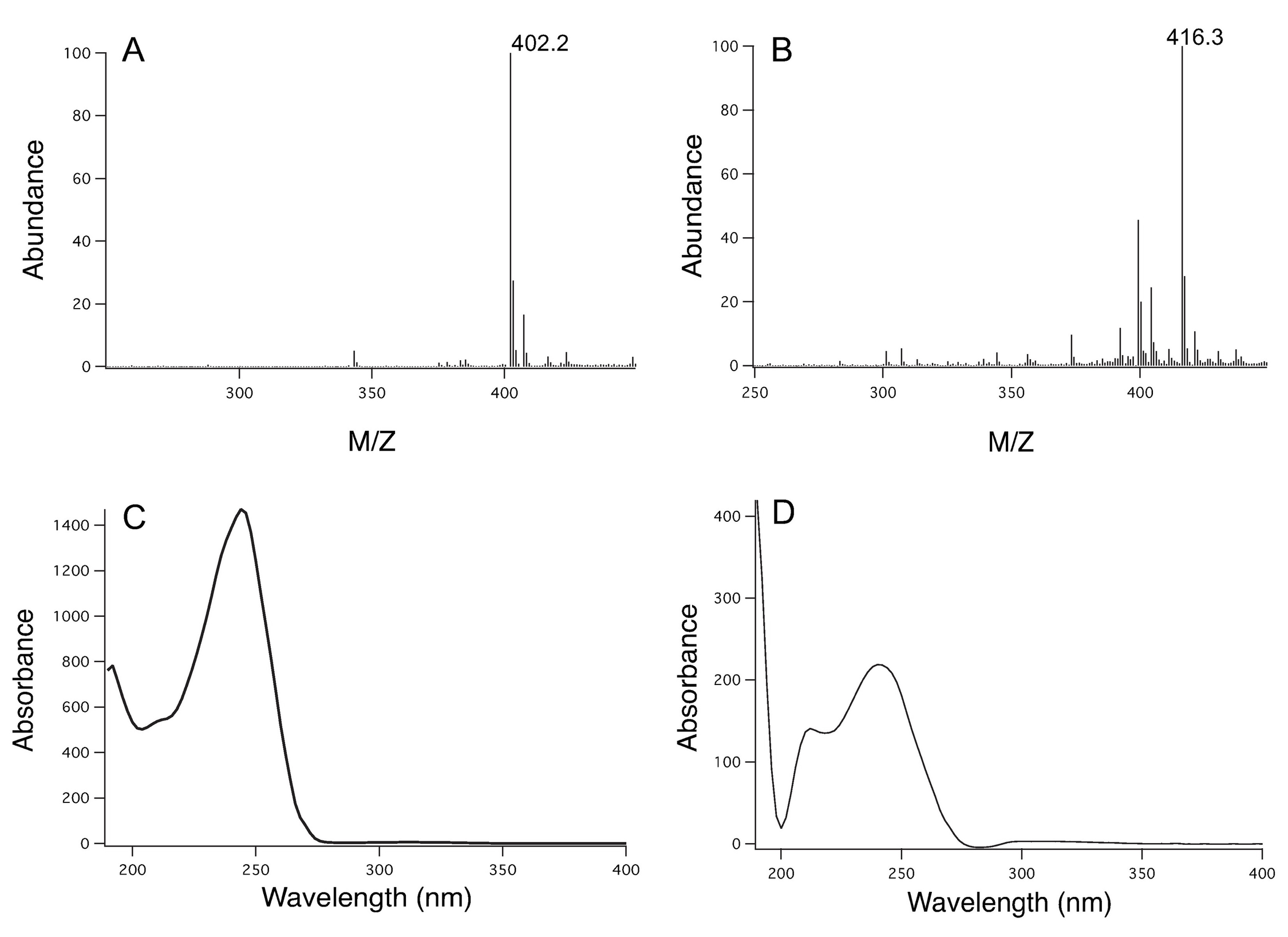

Supplement: S1 Fig — Results for Main Compound 1 shown in panels A and C; Main Compound 2 is shown in panels B and D. (TIF) [file pone.0174816.s003.tif]
